# Poly-*N*-Acetyllactosamine Neo-Glycoproteins as Nanomolar Ligands of Human Galectin-3: Binding Kinetics and Modeling

**DOI:** 10.3390/ijms19020372

**Published:** 2018-01-26

**Authors:** Ladislav Bumba, Dominic Laaf, Vojtěch Spiwok, Lothar Elling, Vladimír Křen, Pavla Bojarová

**Affiliations:** 1 Institute of Microbiology of the Czech Academy of Sciences, Vídeňská 1083, 14220 Prague, Czech Republic; bumba@biomed.cas.cz (L.B.); kren@biomed.cas.cz (V.K.); 2 Laboratory for Biomaterials, Institute for Biotechnology and Helmholtz-Institute for Biomedical Engineering, RWTH Aachen University, Pauwelsstrasse 20, 52074 Aachen, Germany; d.laaf@biotec.rwth-aachen.de (D.L.); l.elling@biotec.rwth-aachen.de (L.E.); 3 Department of Biochemistry and Microbiology, University of Chemistry and Technology Prague, Technická 3, 16628 Prague 6, Czech Republic; Vojtech.Spiwok@vscht.cz

**Keywords:** carbohydrate, galectin-3, galectins in diagnosis, galectins in therapy, glycosyltransferase, surface plasmon resonance, molecular modeling

## Abstract

Galectin-3 (Gal-3) is recognized as a prognostic marker in several cancer types. Its involvement in tumor development and proliferation makes this lectin a promising target for early cancer diagnosis and anti-cancer therapies. Gal-3 recognizes poly-*N*-acetyllactosamine (LacNAc)-based carbohydrate motifs of glycoproteins and glycolipids with a high specificity for internal LacNAc epitopes. This study analyzes the mode and kinetics of binding of Gal-3 to a series of multivalent neo-glycoproteins presenting complex poly-LacNAc-based oligosaccharide ligands on a scaffold of bovine serum albumin. These neo-glycoproteins rank among the strongest Gal-3 ligands reported, with *K*_d_ reaching sub-nanomolar values as determined by surface plasmon resonance. Significant differences in the binding kinetics were observed within the ligand series, showing the tetrasaccharide capped with *N*,*N′*-diacetyllactosamine (LacdiNAc) as the strongest ligand of Gal-3 in this study. A molecular model of the Gal-3 carbohydrate recognition domain with docked oligosaccharide ligands is presented that shows the relations in the binding site at the molecular level. The neo-glycoproteins presented herein may be applied for selective recognition of Gal-3 both on the cell surface and in blood serum.

## 1. Introduction

Galectin-3 (Gal-3) is the only member of the chimeric subgroup of galectins [1] found in vertebrate animals. It is overexpressed in many cancers, e.g., gastric, colorectal, breast tumors, hepatocellular and pancreatic carcinomas, melanomas or glioblastomas. It participates in crucial cancer-related processes: tumorigenesis, metastasis, and neoplasia, angiogenesis, cell adhesion, apoptosis and survival of tumor cells, as well as their immune escape from the host defense system [2,3,4].

In view of all the tumor promoting effects of Gal-3, the design of synthetic inhibitors of Gal-3 represents the principal strategy in the search for new antitumor drugs and precise cancer diagnostics. High-affinity carbohydrate ligands of Gal-3 are of potential interest in numerous biomedical applications [5]. Though β-galactosides are generally recognized as Gal-3 ligands, we recently found that the terminal *N*,*N′*-diacetyllactosamine (LacdiNAc; GalNAcβ1,4GlcNAc) epitope acts as a selective ligand of Gal-3 compared to galectin-1 [6,7]. The LacdiNAc disaccharide specifically occurs in some *O*- and *N*-linked mammalian glycoproteins and has specialized functions [8,9]; it has also been identified as a specific glyco-biomarker in several types of cancers [10,11,12]. Otherwise, it is overexpressed in parasites [13] and other organisms [14]. We demonstrated that the tetrasaccharide composed of a terminal LacdiNAc epitope and an internal LacNAc (GalNAcβ1,4GlcNAcβ1,3Galβ1,4GlcNAc; LacdiNAc-LacNAc) is a superior ligand of Gal-3 when presented in multivalent mode on bovine serum albumin (BSA) as a protein scaffold [15]. In the present work, we evaluate eight oligosaccharide analogs of poly-LacNAc type by surface plasmon resonance studies, of which the LacdiNAc-LacNAc-decorated neo-glycoconjugate exhibits the highest binding affinity in sub-nanomolar range.

Gal-3 is composed of a C-terminal highly conserved carbohydrate recognition domain (CRD) which binds glycan ligands, and an N-terminal non-lectin domain rich in proline and glycine tandem repeats. The N-terminal domain contains a serine phosphorylation site responsible for the oligomerization and cross-linking activity of Gal-3 [16]. The CRD of Gal-3 comprises 135 amino acids, which harbor a specific binding groove of a total of five subsites (A–E) (Figure S1). This binding groove is able to accommodate up to a tetrasaccharide [17]. The galactose unit of lactose (Galβ1,4Glc) or LacNAc (Galβ1,4GlcNAc) binds to the most conserved subsite C whereas the reducing end Glc(NAc) occupies the second most important subsite D; together they form the conserved binding pocket of the binding site (β-strands S4–S6). Subsites A and B extend beyond the C-3 of galactose and constitute the less conserved binding pocket of the CRD (β-strands S1–S3). The non-conserved and generally less defined subsite E reaches beyond the reducing end of the tetrasaccharide and may interact with moieties attached to the C-1 of Glc(NAc) [18,19]. The eight conserved amino acid residues in the CRD of Gal-3 (Arg144, His158, Asn160, Arg162, Asn174, Trp181, Glu184 and Arg186), and Asp148 then coin the binding specificity for particular carbohydrate ligands and provide the main interactions with the bound ligand in the form of hydrogen bonds and van der Waals interactions [20]. In the present paper we show molecular dynamics simulations of LacdiNAc-LacNAc (**3**) and LacNAc-LacNAc (**4**) tetrasaccharides (the best ligands in the series) in the binding site of Gal-3 and compare them with LacdiNAc (**1**) and LacNAc (**2**) disaccharides (the worst ligands in the series).

Gal-3 is monomeric in solution, though small amounts of oligomeric species have been detected at high concentrations [21]. Several mechanisms have been proposed concerning the self-association and oligomerization of Gal-3 subunits or even lattice formation [22] and precipitation upon contact with multivalent ligands such as laminin [23], asialofetuin (ASF) [24], with synthetic multivalent carbohydrates [25] or even complex monovalent glycans such as lacto-*N*-neotetraose [26].

In sum, the present study reveals the binding kinetics of the strongest multivalent neo-glycoprotein ligands of Gal-3 ever reported and the prominent importance of the terminal LacdiNAc epitope in the tetrasaccharide glycan. The sub-nanomolar affinities of ligands to Gal-3 were determined by surface plasmon resonance (SPR). The novel SPR design with immobilized Gal-3-AVI construct (Gal-3 containing AviTag peptide sequence) shown here represents the optimum approach for measuring kinetics with Gal-3 thanks to its fully maintained flexibility. Moreover, the reversed SPR setup with immobilized neo-glycoprotein ligands provides clues pertaining to the Gal-3 oligomerization. Molecular docking of selected glycan ligands in the Gal-3 CRD discloses the relations in the Gal-3 binding site and relevant lectin-ligand interactions. 

## 2. Results

### 2.1. Preparation of functionalized Poly-LacNAc glycans ***1**–**8*** and Neo-Glycoproteins ***9**–**16***

**Figure 1:**
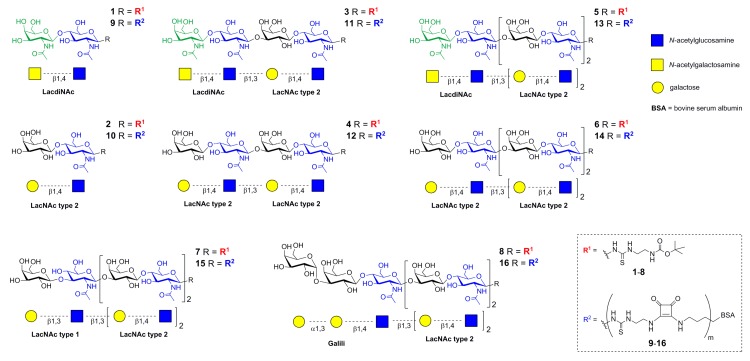
Schematic representation of di- (**1**,**2**), tetra- (**3**,**4**), hexa- (**5**–**7**), and heptasaccharide (**8**) glycans used for covalent modification of BSA to yield respective neo-glycoproteins **9**–**16** [7,15,27].

Oligosaccharide glycans **1**–**8** (Figure 1) carrying a *t-*Boc-protected thioureido linker at the reducing end were prepared as described previously [7,15,27]. The sequential preparative reactions employed a library of tailored glycosyltransferases. The human β4-galactosyltransferase (β4GalT), and the *Helicobacter pylori* β3-*N*-acetylglucosaminyltransferase (β3GlcNAcT) were used for the synthesis of poly-LacNAc type 2 (Galβ4GlcNAc)*_n_*, the mutant human β4-galactosaminyltransferase (β4GalTY284L) for the preparation of LacdiNAc (GalNAcβ4GlcNAc; **1**, **3**, **5**), the *E. coli* β3-galactosyltransferase (β3GalT) for the generation of LacNAc type 1 (Galβ3GlcNAc; **7**), and the murine α3-galactosyltransferase (α3GalT) for the synthesis of Galili (Galα3Galβ4GlcNAc; **8**) (Scheme S1). The structure and purity of glycans **1**–**8** (Figure 1) were confirmed by HPLC-ESI-MS and NMR (Figures S2 and S3) [7,15,27]. 

Neo-glycoproteins **9**–**16** were prepared by conjugating the respective amino-functionalized glycans (**1**–**8**) to free lysine residues of bovine serum albumin (BSA) via two-step amidation using diethyl squarate (3,4-diethoxy-3-cyclobutene-1,2-dione) as described previously [7,15,27] (Scheme S2). The integrity of neo-glycoproteins was checked by SDS-PAGE (Figure S4).

### 2.2. Binding Properties of Glycans ***1**–**8*** and Neo-Glycoproteins ***9**–**16*** to Gal-3 in ELISA Assay

A soluble His-tagged construct of human galectin-3 (Gal-3) was expressed in *E. coli* and purified by immobilized metal-ion affinity chromatography as described before [27]. The binding properties and inhibition parameters of glycans **1**–**8** and respective neo-glycoproteins **9**–**16** were compared using enzyme-linked immunosorbent assays (ELISA).

**Figure 2:**
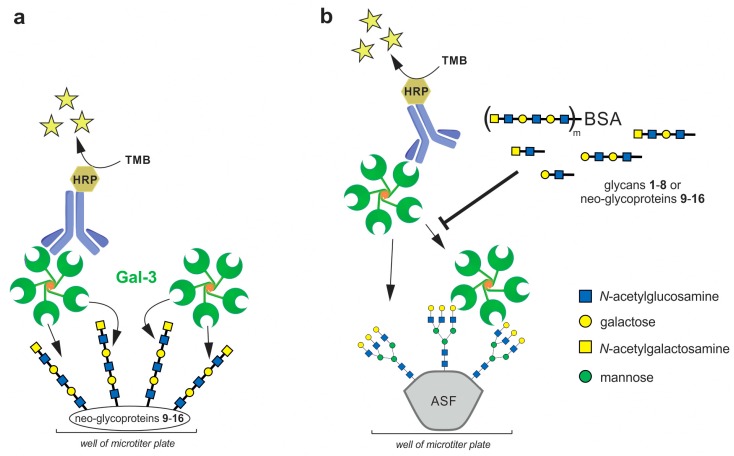
ELISA assays used in the study. (**a**) Direct ELISA assay with immobilized neo-glycoproteins **9**–**16**; (**b**) Competitive ELISA assay using glycans **1**–**8** or neo-glycoproteins **9**–**16** as competing ligands for the inhibition of binding of Gal-3 to immobilized asialofetuin (ASF). The proposed Gal-3 oligomer structure is based on previous reports [25]. Horseradish peroxidase (HRP)-conjugated antibody was used for the detection of bound Gal-3. The HRP converted the added 3,3′,5,5′-tetramethylbenzidine (TMB) to obtain a photometric signal.

To determine the binding affinities between Gal-3 and neo-glycoproteins **9**–**16**, the binding of soluble Gal-3 to the neo-glycoproteins immobilized in microplate wells was quantified by colorimetric immunodetection using anti-His antibody conjugated to horseradish peroxidase (HRP) (Figure 2a) [7,27]. Gal-3 bound neo-glycoproteins in a concentration-dependent manner and the interaction was exclusively conferred by glycan moieties since no binding of Gal-3 to the glycan-free BSA was detected. Apparent dissociation constants (K*_d_*) of the Gal-3/neo-glycoprotein complexes were calculated from a non-linear regression of binding curves. As documented in Table 1, the K*_d_* values were found to range between 30 and 700 nM, except for the neo-glycoproteins **9** and **10**, whose binding affinities were in micromolar concentrations.

To assess the inhibitory potential of glycans **1**–**8** and the respective neo-glycoproteins **9**–**16** towards Gal-3, their capacity to inhibit the binding of Gal-3 to immobilized asialofetuin (ASF) was determined by competitive ELISA inhibition analyses (Figure 2b) [7,15,27]. ASF is a multivalent glycoprotein presenting three triantennary *N*-glycans terminated with LacNAc, which is reported to interact with Gal-3. Here, Gal-3 was incubated with increasing concentrations of the glycans or neo-glycoproteins as competing ligands and the inhibition of Gal-3 binding to immobilized ASF was quantified by colorimetric immunodetection using anti-His antibody conjugated to HRP. Lactose was utilized as a positive control. The respective inhibition constants (IC_50_) were calculated from the non-linear regression of the sigmoidal inhibition curves and they are listed in Table 1.

As shown in Table 1, the inhibitory potencies of individual glycans to Gal-3, expressed as IC_50_, range between 42 and 6.2 µM, with the Galili- (**8**) and especially LacdiNAc capped glycans (**3**, **5**) being the most potent monovalent glycan inhibitors. In comparison, lactose, used as a positive control, had an IC_50_ value of 137 µM. The presence of one (LacNAc, **2**) or two (LacdiNAc, **1**) acetamido groups increased the inhibitory potency up to 4 times compared to lactose. LacdiNAc disaccharide **1** was a slightly less efficient inhibitor than LacNAc (**2**; Figure S5, Table 1). However, LacdiNAc was previously identified as a highly selective inhibitor for Gal-3 compared to other galectins abundant in vivo, in particular galectin-1 [6]. This fact provides an advantage in the development of selective inhibitors for diagnostic and/or specific therapeutic applications.

Neo-glycoproteins **9** and **10** carrying the disaccharides LacdiNAc (**1**) and LacNAc (**2**), respectively, were identified as the least efficient inhibitors (Figure S6). In contrast, the IC_50_ values for neo-glycoproteins **11**–**16** were found to be in low nanomolar range, which reflects a significant increase in their capacity to inhibit Gal-3 binding to ASF. The best neo-glycoprotein ligands in this series are compounds **11** (with tetrasaccharides) and **13** (with hexasaccharides) carrying the terminal LacdiNAc-LacNAc epitope. The terminal LacNAc-LacNAc motif in compounds **12** and **14** is also a good ligand but the inhibition strength is reduced by a factor of 2-3 (*cf*. **11** vs. **12** and **13** vs. **14**), presumably due to the binding preference of the non-conserved pocket of Gal-3 CRD (β-sheets S1–S3) for LacdiNAc as shown further.

The effect of multivalent ligand presentation is reflected in the comparison of the inhibitory potencies of multivalent conjugates with the respective monovalent glycans under consideration of the individual glycan density (relative inhibitory potency per glycan, *r*_p_/*m*, *cf*. Table 1). All neo-glycoproteins except for **1** were shown to exhibit a considerable cluster glycoside effect [28] due to multivalent glycan presentation. Notably, neo-glycoprotein **9** carrying LacdiNAc disaccharide (**1**) did not induce any multivalent effect at all. We conclude that LacdiNAc disaccharide alone, without the LacNAc attachment at the reducing end, is not suitable for a proper interaction with the Gal-3 CRD and for induction of Gal-3 oligomerization. On the contrary, neo-glycoprotein **10** (carrying LacNAc disaccharide) showed a relative inhibitory potency per glycan of 5.8, which confirms the binding specificity of the conserved binding pocket of Gal-3 CRD (β-sheets S4–S6) for LacNAc and the induction of multivalence effect.

### 2.3. Binding Kinetics of Neo-Glycoproteins ***9**–**16*** with Gal-3 Determined by Surface Plasmon Resonance

The kinetics of the interaction of neo-glycoproteins **9**–**16** with Gal-3 were studied by surface plasmon resonance (SPR). This technique measures biomolecular interactions in real-time in a label free environment, where one of the interactants is immobilized to the sensor surface, and the other passes free in solution over the surface as analyte. Several experimental approaches were examined to find optimal conditions for the evaluation of interactions of the tested neo-glycoproteins with Gal-3. To assess ASF binding to immobilized Gal-3, the recombinant His-tagged Gal-3 protein was either covalently immobilized to a carboxylated surface of the GLC (General Layer Chemistry) sensor chip by amine coupling chemistry through its lysine residues or captured by a Ni^2+^-nitrilotriacetate (Ni-NTA) surface through its polyhistidine tag, and ten-fold dilutions of ASF (0.01–10 µM) were injected over the sensor surface. Surprisingly, no SPR response was observed after repeated injections of ASF on the Gal-3 surface in either case, indicating that the covalent immobilization of Gal-3 to the sensor chip completely abolishes its lectin activity. Moreover, the interaction of ASF with Gal-3 captured to the Ni-NTA surface was burdened by a high nonspecific binding of ASF to the sensor chip, which prevented a detailed characterization of the interaction between ASF and Gal-3. Further optimization of the experimental protocols did not improve the quality of the data, showing that the His-tagged Gal-3 protein could not be used as a ligand in the SPR interaction studies using either of these experimental approaches.

**Figure 3:**
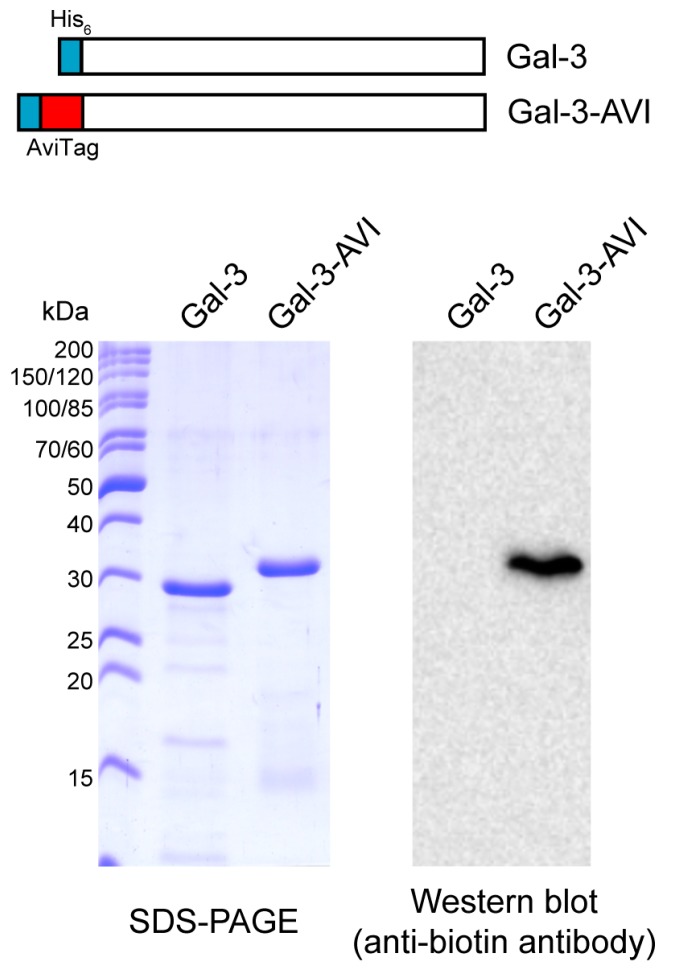
SDS-PAGE and Western blot analyses of the Gal-3-AVI construct.

To overcome the difficulties with Gal-3 immobilization, we prepared a biotinylated version of Gal-3 through in vivo biotinylation of an AviTag peptide that was genetically fused to the N-terminus of Gal-3 (Gal-3-AVI) (Figure 3). The AviTag is a specific 15-amino acid peptide sequence (GLNDIFEAQ**K**IEWHE) that directs a highly targeted enzymatic conjugation of a single biotin molecule to the specific lysine (**K**) residue within the AviTag sequence using biotin ligase (BirA). In contrast to chemical biotinylation, which usually generates heterogeneous products with impaired function, the co-translational biotinylation of the AviTag peptide is site specific and provides a highly homogeneous protein preparation. Moreover, the N-terminal localization of the AviTag peptide provides a favorable orientation of Gal-3-AVI on a streptavidin-coated surface, leaving the *C*-terminal carbohydrate-binding domain of Gal-3 freely accessible for binding interactions. Hence, the Gal-3-AVI protein was co-expressed with BirA in *E. coli* and purified by using the Ni-chelating affinity chromatography. Western blot analysis of the purified Gal-3-AVI showed a single protein band recognized by anti-biotin antibody, indicating the covalent attachment of biotin to Gal-3-AVI (Figure 3). As shown in Table S1, the binding properties of the biotinylated Gal-3-AVI protein were comparable to those of the original Gal-3 construct, documenting that the biotinylated AviTag peptide does not interfere with the lectin activity of Gal-3.

**Figure 4:**
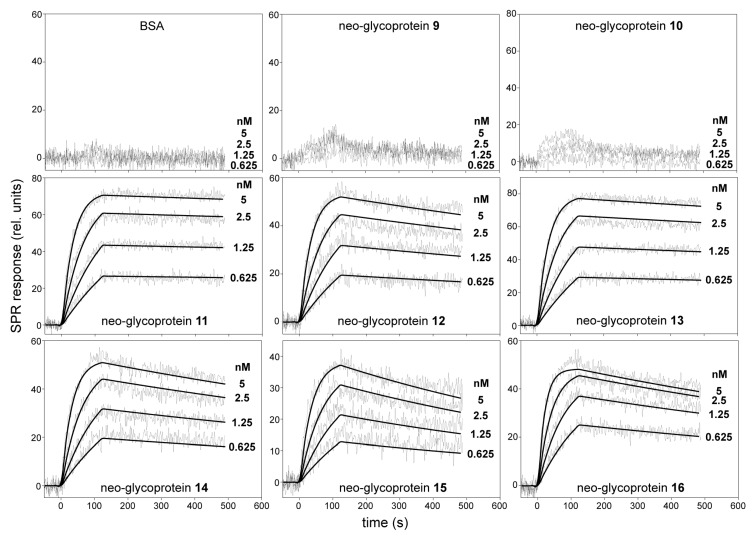
SPR kinetic binding analysis of the interactions between neo-glycoproteins **9**–**16** and immobilized Gal-3-AVI. The neo-glycoproteins at indicated concentrations were injected in parallel over the neutravidin-coated sensor chip coated with the biotinylated Gal-3-AVI at a flow rate of 30 μL/min. The kinetic data were globally fitted by using a 1:1 Langmuir binding model. The fitted curves are superimposed as thin black lines on top of the sensograms. BSA, bovine serum albumin; rel., relative.

For SPR analysis, the biotinylated Gal-3-AVI was captured on a neutravidin-coated sensor chip, and the binding of serially diluted neo-glycoproteins **9**–**16** to immobilized Gal-3-AVI was analyzed. Three coupling concentrations of Gal-3-AVI were tested, so that the effect of mass transfer and the non-specific binding were minimized. As a result, the coupling level of 250 relative units (RU) along with the flow rate of 30 µL/min gave the optimum conditions and these parameters were used for the subsequent experiments. Real time kinetics of the interactions of neo-glycoproteins with immobilized Gal-3-AVI are shown in Figure 4. 

The concentration-dependent binding curves revealed significant differences in the intensity of SPR responses between the neo-glycoproteins carrying the disaccharide (**9**, **10**) and tetra- to heptasaccharide glycans (**11**–**16**). The binding of all tested neo-glycoproteins was exclusively conferred by a glycan moiety since no binding of the glycan-free BSA to Gal-3-AVI was detected. As shown in Figure 4, injection of neo-glycoproteins **9** and **10** at nanomolar concentrations yielded a very low SPR response, indicating that these conjugates carrying disaccharide glycans are rather low-affinity Gal-3 ligands. In contrast, the interaction of neo-glycoproteins **11**–**16** carrying tetra- to heptasaccharide glycans at nanomolar concentrations exhibited typical concentration-dependent binding curves, maintaining both association and dissociation phase of the sensograms. The binding data fitted well to a simple 1:1 Langmuir binding model and the calculated association and dissociation rate constants (*k*_a_ and *k*_d_, respectively) for the interactions between immobilized Gal-3-AVI and the tested neo-glycoproteins are listed in Table 2. The data show that binding affinities (*K*_D_) of Gal-3 to **11**–**16** range in subnanomolar concentrations (10^−10^–10^−11^ M). The LacdiNAc-capped neo-glycoproteins **11** (tetrasaccharide-decorated) and **13** (hexasaccharide-decorated) were identified as the strongest Gal-3 ligands in the series (*K*_D_ of 14 pM and 26 pM, respectively). Such a high binding affinity appears to be due to a very slow dissociation rate, particularly in the **11**/Gal-3 complex (*k*_d_ = 8.5 × 10^−5^·s^−1^) since the association rates of the interaction between the neo-glycoproteins and Gal-3 are comparable. In contrast, the interaction of Gal-3 with neo-glycoprotein **15** carrying a hexasaccharide capped with LacNAc type 1 epitope was characterized by a fast dissociation rate of the complex (*k*_d_ = 1.3 × 10^−3^·s^−1^), yielding a significant decrease of the *K*_D_ value to 270 pM. In comparison, the standard ligand ASF featured a *K*_D_ of 8.3 nM (Table 2 and Figure S7), i.e., it had almost 600-times lower affinity to Gal-3-AVI than **11**. The found *K*_D_ values are by far the lowest values ever determined for Gal-3 by SPR [29,30] and are in the range of affinities of monoclonal antibodies. The *K*_D_ for ASF (positive control) correlates well with the value determined previously by isothermal titration calorimetry (7.14 nM) [31]. Therefore, we conclude that the new approach to immobilization of Gal-3 on the sensor chip via the AviTag-biotin-neutravidin spacer presented here is the most appropriate experimental design for measuring kinetics of the interaction of Gal-3 and carbohydrate ligands thanks to the fully maintained flexibility of Gal-3 on the chip surface. All other experimental approaches give underestimated results, probably due to steric hindrance and partial blocking of the Gal-3 binding site.

To gain further information on the affinity of neo-glycoproteins **9**–**16** to Gal-3, the kinetics of their interaction was determined by SPR in the reversed setup. Neo-glycoproteins **9**–**16** were immobilized at a very low density (~300 RU) on a carboxylated surface of the sensor chip and serial dilutions of His_6_-tag Gal-3, prepared in the same way as for the ELISA experiments, were injected over the sensor surface. Initial binding experiments revealed that low concentrations of Gal-3 (<10 nM) did not yield any SPR response, indicating that binding affinity of Gal-3 to the immobilized neo-glycoproteins is significantly decreased as compared to that obtained for binding of neo-glycoproteins to the immobilized Gal-3-AVI. Higher concentrations of Gal-3 (16–250 nM) resulted in typical concentration-dependent binding curves, except for interaction of Gal-3 with the immobilized disaccharide-carrying neo-glycoproteins **9** and **10** where no binding was detected even at high Gal-3 concentrations (10 μM) (Figure S8). Kinetic parameters of interactions between Gal-3 and neo-glycoproteins **11**–**16** were calculated from global fitting of the binding curves. The data were fitted to several kinetic models, such as 1:1 Langmuir-type, bivalent analyte, heterogeneous ligand and heterogeneous analyte binding models, but only the heterogeneous ligand model provided reasonable fits (“ligand” in this sense refers to the binding partner immobilized on the chip surface). Further optimization of the experimental layout (changing the ligand density, flow rates, etc.) did not improve the quality of the fits and the heterogeneous ligand model was used for final evaluation of the binding curves (Figure S8). This model accounts for binding of an analyte to two ligand species, which may represent either two different molecules or two different (types of) binding sites on the same ligand molecule. Thus, the interaction can be described by complex kinetics involving two separate binding events. The calculated association and dissociation rate constants (*k*_a1_, *k*_d1_ and *k*_a2_, *k*_d2_) are listed in Table S2. The data showed that the interactions of immobilized neo-glycoproteins **11**–**16** with Gal-3 are characterized by a set of two binding affinity constants, where the former (*K*_D1_) range in submicromolar (10^−7^) and the latter (*K*_D2_) in nanomolar (10^−8^) concentrations. The difference lies mainly in the dissociation rate constants (*cf*. *k*_d1_ and *k*_d2_) whereas the association rate constants are rather similar. The *K*_D_ values are very similar throughout the set of neo-glycoconjugates and generally follow the trends observed in ELISA assay and SPR setup with Gal-3-AVI (*cf.* Table 1 and Table 2). However, the binding affinities of immobilized neo-glycoproteins to Gal-3 are much lower than those obtained from the measurements with immobilized Gal-3-AVI, and, most likely, they do not reflect the real interaction data. Moreover, binding of Gal-3 to multivalent glyco-ligands is known to provoke spontaneous Gal-3 oligomerization [24,26], which complicates the use of Gal-3 as an analyte in the SPR studies. Taken together, these data clearly suggest that the use of biotinylated Gal-3-AVI is a much more suitable approach for probing the binding activity of Gal-3 by SPR.

### 2.4. Molecular Dynamics of Disaccharide (***1**,**2***) and Tetrasaccharide (***3**,**4***) Glycans in Gal-3 CRD

We performed molecular dynamics simulations in order to describe the differences in affinities between disaccharide ligands **1** and **2** carried by neo-glycoconjugates **9** and **10**, and ligands **3** and **4** carried by **11** and **12**, which represent the weakest and the strongest pair of ligands in the series, respectively. For this aim, we built a molecular model of Gal-3 CRD based on the experimentally determined crystal structure of Gal-3 CRD in complex with lacto-*N*-neotetraose, Galβ1,4GlcNAcβ1,3Galβ1,4Glc (PDB ID: 4LBN [17]). This is one of the only two known crystal structures of wild-type Gal-3 CRD complexed with an oligosaccharide ligand (PDB ID: 4LBN, 4LBM [17]) and out of the two ligands published, the structure of lacto-*N*-neotetraose is more closely related to glycans **3** and **4**, differing basically in the 2-acetamido group at the reducing-end GlcNAc. So far, all crystallization attempts of Gal-3 were limited only to the carbohydrate binding domain as the N-terminal domain is too flexible and therefore difficult to crystallize [20]. Moreover, the N-terminal domain is known to participate in the formation of the quaternary Gal-3 structure, contributing to the Gal-3 oligomerization and avidity [23], whereas it is less important for the very ligand binding [26]. 

All simulations were done in GROMACS 5.1.3 [32] and the system was simulated in Amber99SB-ILDN force field [33]. Glycan ligand topologies were generated using GLYCAM web server (Glycam 06) [34] and converted to GROMACS by ACPYPE [35]. For the modeling purposes, we used glycans **1**–**4** truncated to the saccharide part (without the C-1 linker) since general force fields used for modeling of organic compounds are not appropriate for carbohydrates, and it was necessary to use a carbohydrate-tailored force field for the Glycam-generated topologies. Generally, it is not excluded to combine a carbohydrate and general force field for one molecule but this procedure is not without risks of possible artefacts, and our preliminary study showed the disadvantages of this approach. 

Our initial attempts for docking of glycans **1**–**4** by PLANTS 1.2 software [36] did not give satisfactory results for the terminal LacNAc domain in substrates **2** and **4** and also for lacto-*N*-neotetraose used as a control ligand, probably due to too many degrees of freedom for the position and rotation of the terminal non-reducing galactose unit. Thus, the protein-glycan complexes acquired by this approach were not suitable as a starting point for molecular dynamics simulations. Therefore, in order to test the binding modes of compounds **1**–**4**, we opted for manual docking of **1**–**4** into the Gal-3 CRD, followed by molecular dynamics simulation (30 ns) for each complex.

**Figure 5:**
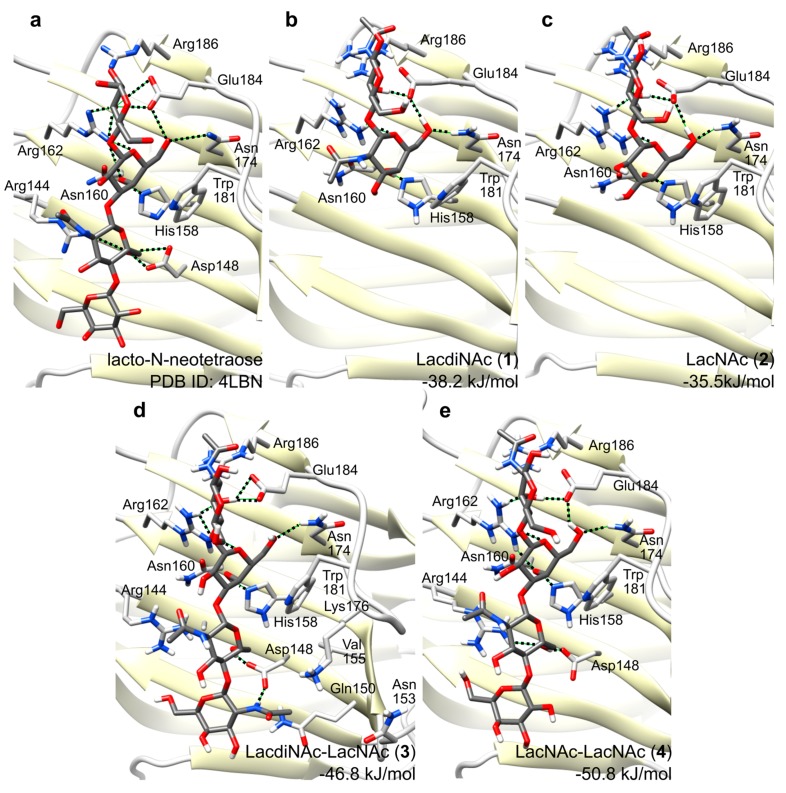
Complexes of glycans **1**–**4** in Gal-3 CRD after 30 ns molecular dynamics simulation. (**a**) Experimentally determined structure of the complex with lacto-*N*-neotetraose (PBD ID: 4LBN) [17]; (**b**) LacdiNAc disaccharide ligand **1**; (**c**) LacNAc disaccharide ligand **2**; (**d**) LacdiNAc-LacNAc tetrasaccharide ligand **3**; (**e**) LacNAc-LacNAc tetrasaccharide ligand **4**. The amino acid residues important for the glycan binding are depicted in stick representations, with oxygens in red and nitrogens in blue.

All four simulated complexes were stable during simulations. The main glycan-protein interactions, such as the stacking interaction to Trp181, and the hydrogen bond to His158, were retained throughout the whole simulations. Figure 5 shows snapshots at the end of respective simulations for glycans **1**–**4** with Gal-3 CRD (PDB ID: 4LBN), and for lacto-*N*-neotetraose for comparison. Both disaccharidic ligands **1** and **2** bind to the binding groove (conserved binding pocket of subsites C, D) in perfect agreement with the experimentally determined complexes with lactose (PDB ID: 3ZSJ [37]), lacto-*N*-neotetraose (PDB ID: 4LBN [17]), and lacto-*N*-tetraose (PDB ID: 4LBM [17]). The simulation shows that the *N*-acetamido moiety at the terminal galactoside residue in **1** (GalNAcβ1,4GlcNAc) does not form any direct hydrogen bonds with the protein and it rather protrudes in the direction out of the binding site (Figure 5). This may explain the fact that binding affinities of disaccharides **1** and **2** are comparable as determined in ELISA assays (Table 1). A higher affinity for LacNAc over lactose is caused by stacking of the reducing-end GlcNAc *N*-acetamido moiety against the side chain of Arg186.

The binding free energies for glycan ligands **1**–**4** were predicted by Linear Interaction Energy method as −38.2, −35.5, −46.8 and −50.8 kJ/mol, respectively. These values tend to overestimate binding, and an accurate prediction would require tuning of the scaling factors of Linear Interaction Energy method using a large series of ligands; nevertheless, they follow the trend of binding free energies with approx. 4 kJ/mol accuracy. Binding of **3** and **4** involves additional interactions absent in the binding of **1** or **2**, which occur between the non-reducing end disaccharide GalNAcβ1,4GlcNAc bound in the second, less conserved binding pocket (subsites A, B), and mainly with Arg144, Asp148 as well as water-mediated interactions. This fact explains a significantly higher affinity of Gal-3 CRD for the tetrasaccharide ligands. This is also reflected in the binding free energies, which are significantly lower for tetrasaccharides **3** and **4** compared to disaccharides **1** and **2**. The *N*-acetamido moiety of GalNAc in **3** binds into the pocket formed by residues Gln150, Asn153, Val155 and Lys176 and it makes a hydrogen bond with one oxygen of Asp148 whereas the second oxygen binds the 6-hydroxyl group of the GlcNAc residue. These additional interactions may explain a higher affinity towards **3** relative to **4**, which does not contain this acetamido moiety.

## 3. Discussion

The present study on various structural aspects of multivalent poly-LacNAc-type glycans with respect to the binding and inhibition of Gal-3 clearly highlights the tetrasaccharide LacdiNAc-LacNAc (**3**) as the optimum ligand for Gal-3. For efficient Gal-3 binding, the interaction with longer oligosaccharides is required as suggested previously [17,38,39]. Not only are longer glycans sterically more accessible for lectin interaction [7] but, more importantly, the unique poly-LacNAc-type pattern specifically binds to both the conserved and non-conserved parts of the binding groove on the Gal-3 CRD. Thus, both the first (β-strands S4–S6) and second (β-strands S1–S3) binding pockets are occupied by LacNAc or related structures (Figure 1 and Table 1) as hypothesized previously [17,27,39,40]. The advantage of the tetrasaccharide ligand **4** is in the optimum combination of binding of the LacNAc part in the conserved first binding pocket (β-strands S4–S6) and of LacdiNAc part in the non-conserved second binding pocket of the CRD (β-strands S1–S3). The terminal GalNAc moiety of **3** exhibits additional hydrogen bonding with Asp148, which apparently stabilizes glycan **3** in the Gal-3 binding groove relative to **4**. Binding kinetics data clearly support this theory since they show that the difference between the LacdiNAc-capped glycan **3** and its LacNAc counterpart **4** is not given by a better recognition of the glycan but by its better stabilization in the binding site (*k*_a_ rate constants are similar for both neo-glycoproteins **11** and **12** whereas *k*_d_ is almost 5 times lower for **11**; *cf*. Table 2).

A detailed comparison of found *K*_D_ values (Table 2) reveals that the binding properties of the neo-glycoproteins carrying the same epitope are relatively similar for the tetrasaccharide- and hexasaccharide-carrying neo-glycoproteins (*cf*. Table 2, **11** vs. **13**, and **12** vs. **14**). Gal-3 is stated to primarily recognize the internal LacNAc motifs [41,42]. Therefore, the LacdiNAc-LacNAc-LacNAc hexasaccharide on neo-glycoprotein **13** can be bound in two modes, either involving the terminal LacdiNAc-LacNAc or only the internal LacNAc-LacNAc. We suggest that this fact accounts for the 1.8-times lower affinity (*K*_D_) of Gal-3 to **13** compared to **11**, since **13** allows also the less efficient LacNAc-LacNAc binding mode.

Another noteworthy result is the huge difference in binding kinetics observed between the individual SPR setups. The binding kinetics using immobilized Gal-3-AVI is clearly described by 1:1 Langmuir-type model, correctly mirroring the fact that the immobilized Gal-3 maintained its monomeric form throughout the interaction. In contrast, the reversed setup with Gal-3 in solution states a much more complex binding kinetics described by heterogenous ligand model. When consulting the literature, a similar complex behavior was observed by SPR when Gal-3 interacted with some multivalent macromolecular ligands such as laminin [43]. Laminin is a complex glycoprotein carrying poly-LacNAc chains [44]. In contrast, SPR with immobilized monovalent low-molecular ligands such as lactosides fit the simple 1:1 binding model [45,46]. Taking into account that complex, especially multivalent glyco-ligands are known to incite Gal-3 oligomerization by several described mechanisms [24,26], we hypothesize that the heterogenous nature of the interaction of immobilized neo-glycoconjugates with Gal-3 in solution might reflect the oligomerization of Gal-3 monomers, triggered by binding to these multivalent ligands. A more detailed experimental analysis of this process is yet to be performed. The considerably weaker interaction found in the reversed setup (nevertheless, comparable with other SPR setups for Gal-3 found in the literature) may be caused by a partial steric obstruction of some glycans presented on the immobilized multivalent BSA scaffold, as a result of the non-directed (random) immobilization procedure. In contrast, our novel SPR setup using Gal-3-AVI exploits the advantage of the fully flexible attachment via biotin-AviTag.

## 4. Materials and Methods

### 4.1. Synthesis of Glycans ***1**–**8*** and Neo-Glycoproteins ***9**–**16***

The recombinant glycosyltransferases used in this study were produced in *E. coli* and purified by affinity chromatography as described earlier [15,27,47,48,49]. Functionalized glycans **1**–**8** were synthesized from (*tert*-butoxycarbonylamino)ethylthioureidyl 2-acetamido-2-deoxy-β-d-glucopyranoside [50] in high-yielding sequential preparative reactions employing a series of glycosyltransferases as described previously [27,51]. The synthetic protocol (Scheme S1) for the preparation of **1**–**8** can be found in the Supplementary Materials. The synthesis of neo-glycoproteins **9**–**16** was performed as reported earlier [7,15,27]. Briefly, the amino-functionalized glycans **1**–**8** were deprotected and conjugated with 3,4-diethoxy-3-cyclobutene-1,2-dione (diethyl squarate) to yield squarate monoamide esters, which were isolated by preparative HPLC. Then, the amidation of free lysine residues of BSA with and subsequent purification by preparative HPLC yielded neo-glycoproteins **9**–**16.** Detailed information on syntheses including Scheme S2 can be found in the Supplementary Materials.

### 4.2. Protein Production

Asialofetuin was purchased from Sigma-Aldrich (Steinheim, Germany), dissolved in phosphate-buffered saline (PBS) at 1 mg/mL and stored at aliquots at −20 °C. The recombinant human His_6_-tagged galectin-3 protein (Gal-3) was produced and purified as described earlier [15,27,30,49]; the experimental details are included in the Supplementary Materials. For the expression of the biotinylated Gal-3-AVI protein, the AviTag sequence (GLNDIFEAQKIEWHE) was genetically introduced into the plasmid construct encoding the His_6_-tagged Gal-3 (pETDuet1-Gal-3). A pair of synthetic oligonucleotides (5′-GATCCGGGTCTGAACGACATCTTCGAGGCTCAGAAAATCGAATGGCACGAAGGTGGATCTGGTGGATCTGCG-3′ and 5′-AATTCGCAGATCCACCAGATCCACCTTCGTGCCATTCGATTTTCTGAGCCTCGAAGATGTCGTTCAGACCCG-3′) was annealed to give the double-stranded DNA fragment carrying the BamHI and EcoRI overhangs and cloned into the BamHI/EcoRI-cleaved pETDuet1-Gal-3 vector. The expression of the biotinylated Gal-3-AVI protein was performed in *E. coli* BL21 (λDE3) transformed with the pETDuet1-Gal-3 vector along with an IPTG inducible plasmid containing the *birA* gene. The cells were grown in mineral M9 medium supplemented with glycerol (20 g·L^−1^), yeast extract (20 g·L^−1^), 150 µg·mL^−1^ ampicillin and 10 µg·mL^−1^ chloramphenicol to an optical density of 0.6 at 600 nm, induced with 1 mM isopropyl 1-thio-β-d-galactopyranoside (IPTG) in the presence of 50 μM d-biotin (Sigma), and grown for additional 4 h at 37 °C. The in vivo biotinylated His_6_-tagged Gal-3 protein was purified by Ni^2+^-chelating affinity chromatography analogously to the original construct as described in the Supplementary Materials. Protein concentrations were determined by Bradford assay using a calibration with bovine serum albumin. The molecular weight of Gal-3 and Gal-3-AVI was determined by amino acid sequence to be 28,023 and 30,550 Da, respectively.

### 4.3. ELISA Assays with Gal-3

The affinity of Gal-3 for neo-glycoproteins **9**–**16** was determined using ELISA as reported previously [15,27,30,49]. For the immobilization of the respective neo-glycoproteins or non-modified BSA (negative control), we used F16 Maxisorp NUNC-Immuno Modules (Thermo Scientific, Roskilde, Denmark). Per well, an amount of 5 pmol was incubated overnight at a working concentration of 0.1 µM (PBS). Then the wells were blocked with bovine serum albumin (2% *w*/*v*) diluted in PBS (1 h, room temperature). Afterwards, recombinant Gal-3 in varying concentration (total volume 50 µL) was added and incubated for 1 h. Detection of bound Gal-3 was achieved using anti-His_6_-IgG1 antibody from mouse conjugated with horseradish peroxidase (Roche Diagnostics, Mannheim, Germany) diluted in PBS (1:2000, 50 µL, 1 h, room temperature). TMB One (Kem-En-Tec, Taastrup, Denmark) substrate solution was utilized to initiate reaction of IgG-conjugated peroxidase. The reaction was stopped by adding 3 M hydrochloric acid (50 µL). The binding signal of bound galectin was measured with a spectrophotometer (Spectra Max Plus, Molecular Devices, Sunnyvale, CA, USA) at an optical density of 450 nm. Obtained data were analyzed using SigmaPlot 10 software (Systat Software GmbH, Erkrath, Germany). 

In the competitive ELISA design, the F16 Maxisorp NUNC-Immuno Modules (Thermo Scientific, Roskilde, Denmark) were coated overnight with ASF (Sigma Aldrich, Steinheim, Germany; 0.1 µM in PBS, 50 µL, 5 pmol per well) and blocked with BSA (2% *w*/*v*) diluted in PBS (1 h, room temperature). Afterwards, a mixture of the respective compound **1**–**16** in varying concentrations together with Gal-3 (total volume 50 µL; 5 µM final Gal-3 concentration) were added and incubated for 1 h. Detection of bound Gal-3 and data analysis were performed as described above.

### 4.4. Surface Plasmon Resonance Measurements

SPR measurements were performed at 25 °C using a set of sensor chips (GLC, NLC, HTE) mounted on a ProteOn XPR36 Protein Interaction Array System (Bio-Rad, Hercules, CA, USA ) as described previously [52]. Briefly, Gal-3-AVI protein was diluted to a final concentration of 5 µg·mL^−1^ in PBS and captured on a neutravidin-coated NLC sensor chip (Bio-Rad) at a flow rate of 30 µL·min^−1^. Neo-glycoproteins **9**–**16**, used here as analytes, were serially diluted in the running buffer (PBS supplemented with 0.005% Tween 20), and injected in parallel over the immobilized Gal-3-AVI at a flow rate of 30 µL·min^−1^. The Gal-3-AVI surface was regenerated by a washing step with 50 mM HCl for 60 s. For the reversed experimental setup, the neo-glycoproteins **9**–**16** were immobilized on the GLC sensor chip by amine coupling chemistry and serial dilutions of Gal-3 were injected over the sensor surface. The correction of the sensograms for sensor background was performed by the interspot referencing procedure, which utilizes the sites on the 6 × 6 array without exposure to the immobilization of ligand but only to the flow of analyte). Furthermore, a ‘‘blank’’ injection was used for substracting the value for analyte. The global analysis of data was done by fitting the binding curves using 1:1 Langmuir-type and heterogeneous ligand binding models. The Langmuir-type model presumes that ligand (L) and analyte (A) interact together under the direct formation of the final complex (AL):

$$\;A+L\;\overset{ka,kd\;}{↔}\;AL$$

where *k*_a_ stands for the association rate constant, and *k*_d_ for the dissociation rate constant. Heterogeneous ligand models accounts for the presence of ligands that bind one analyte in two separate binding events:

$$\;A+L1\;\overset{ka1,kd1\;}{↔}\;AL1$$

$$\;A+L2\;\overset{ka2,kd2\;}{↔}\;AL2$$

where *k*_a1_ and *k*_d1_, and *k*_a2_ and *k*_d2_ represent the association and dissociation rate constants of the former and the latter binding, respectively. The equilibrium dissociation constant, *K*_D_, was determined as a ratio between the dissociation and association rate constants:

$$\;K_{D}=\frac{k_{d}}{k_{a}}\;$$

### 4.5. Molecular Dynamics Simulations

The molecular model of Gal-3 CRD, used for molecular dynamics simulations, was constructed from the experimentally determined structure of Gal-3 CRD in complex with lacto-*N*-neotetraose (PDB ID: 4LBN [17]). All simulations were done in GROMACS 5.1.3 [32]. The system was simulated in Amber99SB-ILDN force field [33]. Ligand topologies were generated using GLYCAM web server (Glycam 06) [34] and converted to GROMACS by ACPYPE [35]. Lengths of all covalent bonds were constrained in all simulations. Simulation step was set to 2 fs. Electrostatics was calculated using particle-mesh Ewald method [53] and temperature was controlled by Parrinello-Bussi thermostat [54].

Ligands were docked into the binding site by manual alignment of respective carbohydrate moieties and adjustment of ligand torsion in UCSF Chimera [55]. Each system was minimized in vacuum (approximately 15–25,000 steps). Then it was solvated and neutralized by replacing the randomly chosen water molecules by chloride anions. Each system contained a monomer of Gal-3 CRD, a glycan ligand, 8453–9046 TIP3P water molecules and 4 chloride counterions. Then, it was minimized again (approximately 10–15,000 steps), followed by restrained NVT (constant number of particles, volume and temperature) simulations at 10, 50 and 100 K; 10 ps each. Furthermore, restrained NPT (constant number of particles, pressure and temperature) simulation of 100 ps at 300 K and restrained NVT simulation of 1.1 ns at the same temperature were performed. Harmonic position restraints with a force constant of 1000 kJ·mol^−1^·nm^−2^ were used in all restrained simulations. They were followed by unrestrained simulations (30 ns) at 300 K.

Binding free energies were predicted by the Linear Interaction Energy method [56] based on the last 10 ns of each simulation and 10 ns simulations of unbound solvated ligands. Scaling factors for Lennard-Jones and electrostatic energies were 0.181 and 0.3, respectively.

## 5. Conclusions

The present paper discloses the binding kinetics of the strongest multivalent ligand of Gal-3 ever reported: a BSA-based neo-glycoprotein carrying LacdiNAc-LacNAc tetrasaccharides. This conjugate exhibits pM affinities to Gal-3 as determined by SPR in a novel setup with Gal-3 immobilized via Avi-biotin-neutravidin tag. This setup allows the determination of binding constants comparable to the values in solution as shown by isothermal titration calorimetry. Through molecular dynamics studies, we identified the interactions of this tetrasaccharide in the Gal-3 binding site and proposed possible explanations for its high affinity. The results herein shed more light on the interaction of poly-LacNAc-type glycoconjugates with Gal-3 and draft new pathways in the direction of developing specific efficient inhibitors of Gal-3 applicable in cancer-related diagnosis and therapies.

## Figures and Tables

**Table 1:** Binding properties of glycans **1**–**8** and respective neo-glycoproteins **9**–**16** in ELISA assay.

Neo-Glycoprotein (Respective Glycan) ^a^	M_w_ (kDa)	*m* ^b^	IC_50_ Glycan (µM) ^c^	IC_50_ Neo-Glycoprotein (nM)	*r*_p_^d^	*r*_p_/*m*^e^	*K*_d_ Neo-Glycoprotein (nM)
**9** (glycan **1**)	76.4	17	42 ± 2	2026 ± 147	20.7	1.2	6290 ± 530
**10** (glycan **2**)	78.2	18	36 ± 1	344 ± 38	104.7	5.8	4780 ± 1240
**11** (glycan **3**)	87.4	21	7 ± 1 ^f^	11 ± 2	642.9	30.6	30 ± 4 ^f^
**12** (glycan **4**)	84.7	19	13 ± 3 ^f^	31 ± 1	419.4	22.1	300 ± 60 ^f^
**13** (glycan **5**)	86.6	16	6.20 ± 0.02 ^g^	37 ± 7 ^g^	169.9	10.6	76 ± 19 ^g^
**14** (glycan **6**)	86.6	17	20.1 ± 0.1 ^g^	76 ± 8 ^g^	263.4	15.5	350 ± 110 ^g^
**15** (glycan **7**)	85.9	17	12.5 ± 0.1 ^g^	212 ± 22 ^g^	58.9	3.5	700 ± 100 ^g^
**16** (glycan **8**)	89.6	18	8.4 ± 0.1 ^g^	65 ± 4 ^g^	128.2	7.1	290 ± 90 ^g^

^a^ The numbers in parentheses indicate the compound numbers of respective glycans bound on the neo-glycoprotein; ^b^ Average number of glycans per BSA molecule; ^c^ *cf*. IC_50_ (lactose) = 137 ± 27 µM; ^d^ Relative potency, i.e., IC_50_ (monovalent glycan)/IC_50_ (multivalent neo-glycoprotein); ^e^ Relative potency per glycan bound on BSA; ^f^ The data were adopted from our previous study [15]; ^g^ The data were adopted from our previous study [27].

**Table 2:** Kinetic affinity constants for the interactions of neo-glycoconjugates **9**–**16** with immobilized Gal-3-AVI.

Compound	Attached Glycan ^a^	*k*_a_ (M^−1^·s^−1^)	*k*_d_ (s^−1^)	*K*_D_ (M)
**9**	LacdiNAc	N.D.	N.D.	N.D.
**10**	LacNAc	N.D.	N.D.	N.D.
**11**	LacdiNAc-LacNAc	(5.9 ± 1.3) × 10^6^	(8.5 ± 2.5) × 10^−5^	(1.4 ± 0.4) × 10^−11^
**12**	LacNAc-LacNAc	(6.2 ± 2.1) × 10^6^	(4.2 ± 3.1) × 10^−4^	(6.8 ± 3.9) × 10^−11^
**13**	LacdiNAc-LacNAc-LacNAc	(5.8 ± 2.2) × 10^6^	(1.5 ± 0.4) × 10^−4^	(2.6 ± 0.9) × 10^−11^
**14**	LacNAc-LacNAc-LacNAc	(7.8 ± 2.9) × 10^6^	(5.1 ± 2.8) × 10^−4^	(6.5 ± 2.6) × 10^−11^
**15**	LacNAc type 1-LacNAc-LacNAc	(4.9 ± 1.8) × 10^6^	(1.3 ± 0.3) × 10^−3^	(2.7 ± 1.0) × 10^−10^
**16**	Galili-LacNAc-LacNAc	(9.3 ± 3.1) × 10^6^	(6.3 ± 2.0) × 10^−4^	(6.8 ± 3.5) × 10^−11^
ASF	Positive control standard	(4.8 ± 1.9) × 10^4^	(4.0 ± 1.7) × 10^−4^	(8.3 ± 3.1) × 10^−9^

^a^ Structures of glycans attached to respective neo-glycoproteins are depicted in Figure 1. N.D., not determined.

## Supplementary Materials

Supplementary materials can be found at http://www.mdpi.com/1422-0067/19/2/372/s1.

## Abbreviations

ASF	Asialofetuin
BSA	Bovine serum albumin
CRD	Carbohydrate recognition domain
ELISA	Enzyme-linked immunosorbent assay
ESI-MS	Electrospray ionization mass spectrometry
Gal-3	Galectin-3
Gal-3-AVI	Galectin-3 containing AviTag peptide sequence
HPLC	High-performance liquid chromatography
IC_50_	Half maximal inhibitory concentration
LacNAc	*N*-Acetyllactosamine (β-d-Gal-(1→4)-d-GlcNAc)
LacdiNAc	*N*,*N′*-Diacetyllactosamine (β-d-GalNAc-(1→4)-d-GlcNAc)
NMR	Nuclear magnetic resonance
SDS-PAGE	Sodium dodecyl sulfate polyacrylamide gel electrophoresis
SPR	Surface plasmon resonance
TMB	3,3′,5,5′-tetramethylbenzidine
