# Supplementary material for: Poly-N-Acetyllactosamine Neo-Glycoproteins as Nanomolar Ligands of Human Galectin-3: Binding Kinetics and Modeling
Source: Int J Mol Sci. 2018 Jan 26;19(2):372. doi: 10.3390/ijms19020372 (PMC5855594; doi:10.3390/ijms19020372)
Supplement: Supplementary file 1 [file ijms-19-00372-s001.pdf]

International Journal of *Molecular Sciences*

MDPI

*Supplementary Materials*

# Poly-*N*-Acetyllactosamine Neo-Glycoproteins as Nanomolar Ligands of Human Galectin-3: Binding Kinetics and Modeling

**Ladislav Bumba, Dominic Laaf, Vojtěch Spiwok, Lothar Elling, Vladimír Křen and Pavla Bojarová**

**Contents**

1. Molecular model of Gal-3 carbohydrate recognition domain (PDB ID: 4LBN) (Figure S1)
2. Synthesis of functionalized glycans **1–8** (Scheme S1)
3. Synthesis and characterization of neo-glycoproteins **9–16** (Scheme S2, Figures S2, S3, S4)
4. Production of soluble recombinant His$_6$-tagged galectin-3
5. ELISA-type binding assays, binding curves (Figures S5, S6)
6. Binding and inhibition properties of Gal-3-AVI (Table S1)
7. SPR sensograms and kinetic data table (Figures S7, S8, Table S2)

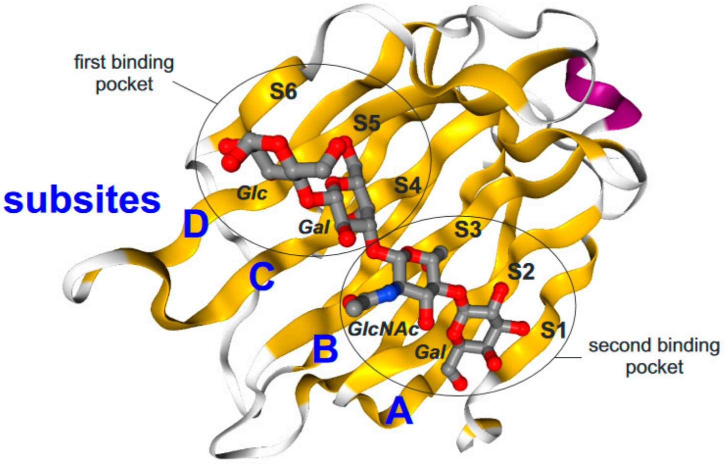


**Figure S1**. Molecular model of Gal-3 carbohydrate recognition domain (CRD) (PDB ID: 4LBN), adapted from the literature [1-3]. Lacto-*N*-neotetraose (Galβ1,4GlcNAcβ1,3Galβ1,4Glc) occupies both the first binding pocket (β-strands S4-S6) and the second binding pocket (β-strands S1-S3).

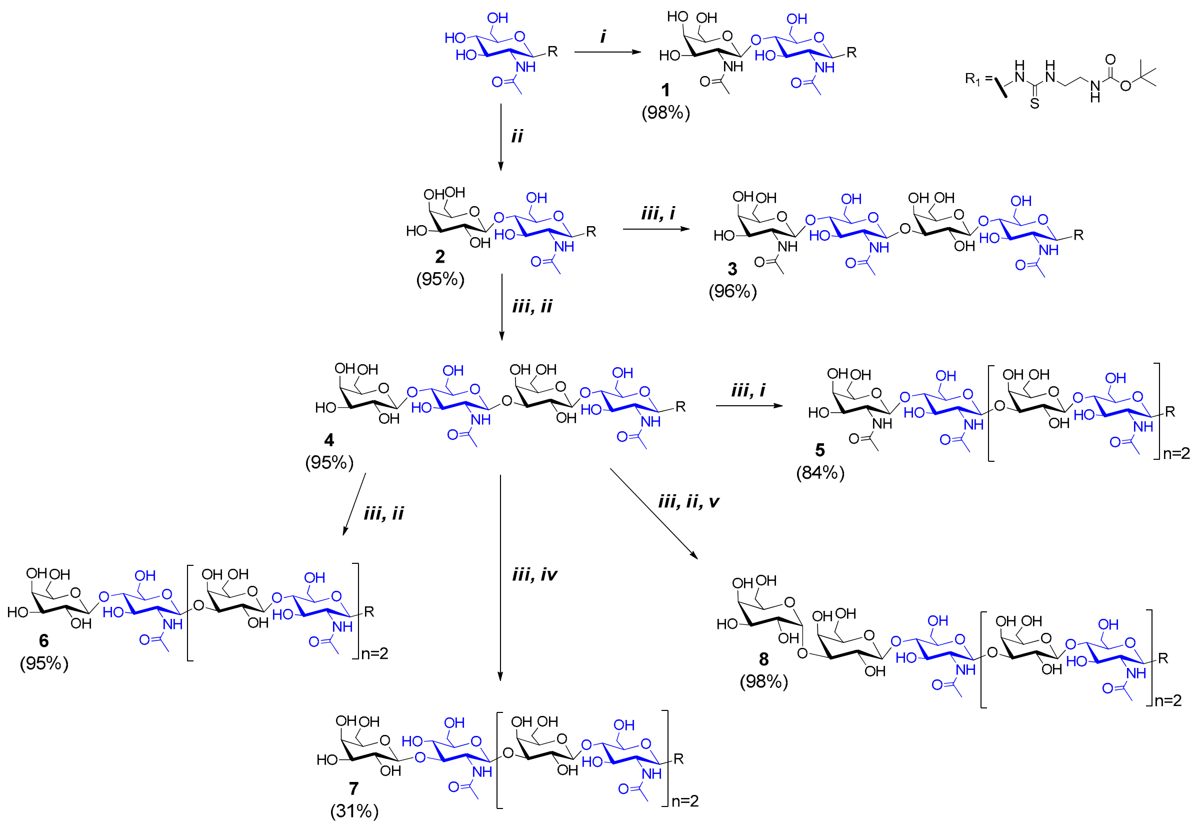


**Scheme S1.** Enzymatic synthesis of glycans **1**-**8** using mutant human β4-galactosyltransferase (β4GalTY284L, ***i***), human β4-galactosyltransferase (β4GalT, ***ii***), *Helicobacter pylori* β3-*N*-acetylglucosaminyltransferase (β3GlcNAcT, ***iii***), *E. coli* β3-galactosyltransferase (β3GalT, ***iv***) and murine α3-galactosyltransferase (α3GalT, ***v***) according to previous reports [3-6]. All reactions were supplemented with the appropriate glycosyl acceptors (5 mM), the respective activated nucleotide sugar donors (1.5-3.0-fold molar excess of UDP-GalNAc, UDP-Gal, UDP-GlcNAc), equimolar divalent cations (***i***, ***ii*** and ***v*** = $Mn^{2+}$; ***iii*** and ***iv*** = $Mg^{2+}$) and alkaline phosphatase (degradation of UDP, which may inhibit glycosyltransferase reaction). The reactions were performed in aqueous buffers according to the pH optimum of each individual enzyme (***i***, ***iii*** and ***iv*** = 100 mM HEPES [pH 7.6]; ***ii*** = 100 mM MOPS [pH 6.8]; ***v*** = 100 mM MOPS [pH 6.0]) at 30 °C without shaking. After proceeded reaction, samples were heated (95 °C, 5 min), centrifuged (13,400 rpm, 10 min) and analyzed by reversed-phase HPLC (Dionex HPLC system, column: MultoKrom C18 250 mm × 4 mm; CS-Chromatographie, Langerwehe, Germany) in 15 % (*v*/*v*) acetonitrile in double distilled $H_20$ supplemented with 0.1 % (*v*/*v*) formic acid at a flow rate of 1 mL/min. Preparative purification was performed by solid-phase extraction (Sep-Pak C18 1/3/6cc Vac Cartridges, Waters Corporation) according to manufacturer's instructions. Alternatively, products were purified by preparative HPLC using the above conditions with MultoKrom C18 (250 mm × 20 mm, CS-Chromatographie, Langerwehe, Germany) and an adjusted flow rate of 25 mL/min.

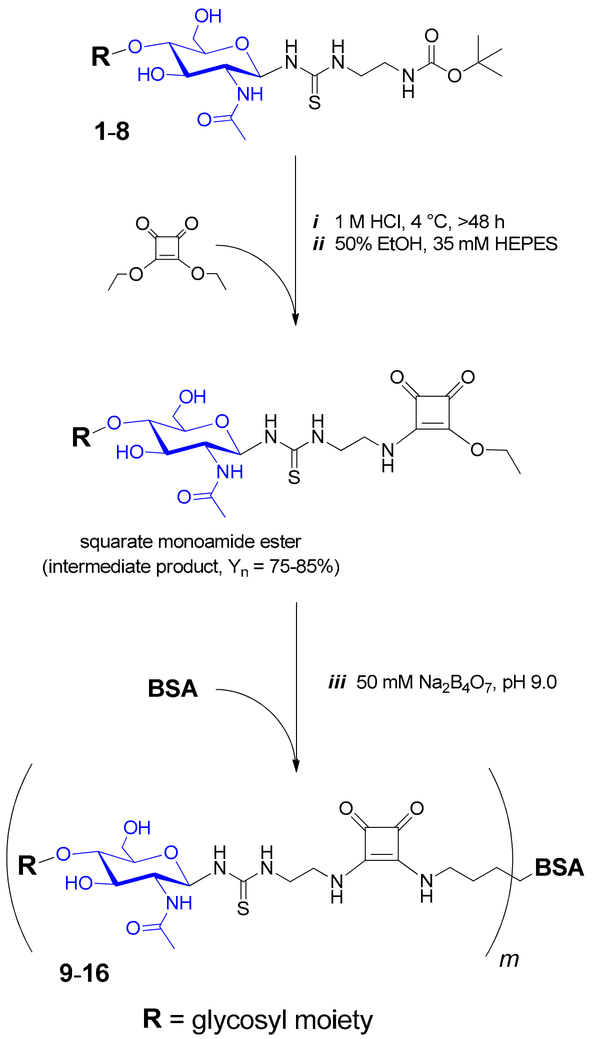


**Scheme S2.** Synthesis of multivalent neo-glycoproteins **9–16** using diethyl squarate as a homobifunctional linker. Oligosaccharides **1–8** were deprotected under acidic conditions as indicated. The resulting primary amine was reacted with a four-fold molar excess of diethyl squarate and triethylamine in 50% aqueous ethanol (23 °C, 18 h, 400 $min^{-1}$ shaking frequency). The purification of squarate monoamide ester intermediate products from the crude reaction was accomplished by preparative HPLC using MultoKrom C18 (250 mm × 20 mm, CS-Chromatographie, Langerwehe, Germany) and an adjusted flow rate of 12.5 mL/min [3,4,6]. Pure intermediate products were mixed with bovine serum albumin (BSA, 0.06 mM) in 50 mM $Na_2B_4O_7$ buffer (pH 9.0). After 7 days of incubation at 23 °C (400 $min^{-1}$ shaking frequency), the resulting neo-glycoproteins **9–16** were harvested by centrifugation using VivaSpin® 500 (Sartorius Stedim Biotech, Göttingen, Germany). The modification density (*m*) of neo-glycoproteins **9–16** was determined by TNBSA-assay [4]. Refer to Scheme S1 for composition of glycosyl moiety residues. Neo-glycoprotein **9** carries glycans **1**, neo-glycoprotein **10** is decorated with glycan **2** and so forth (as also indicated in Table 1 in the main text).

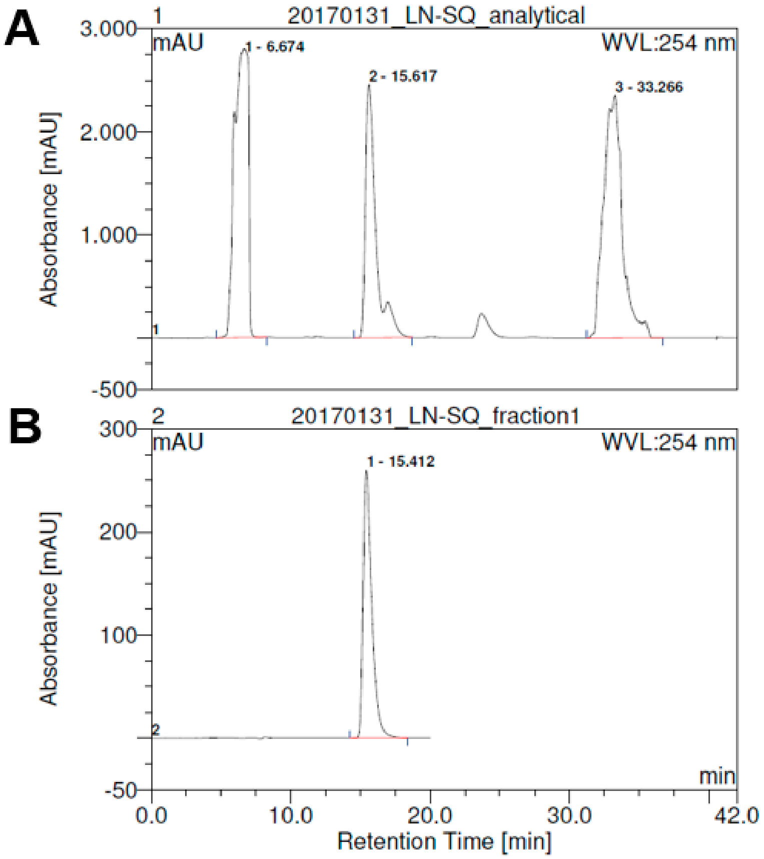


**Figure S2.** LacNAc-squarate monoamide ester before (**A**) and after purification by preparative HPLC (**B**). The reaction was monitored by reversed-phase HPLC (Dionex HPLC system, column: MultoKrom C18 250 mm × 4 mm [CS-Chromatographie, Langerwehe, Germany], 15% (v/v) acetonitrile in MilliQ-$H_2$0, a flow rate of 0.5 mL/min). Preparative purification of squarate monoamide ester was performed by preparative HPLC under the same conditions using MultoKrom C18 (250 mm × 20 mm, CS-Chromatographie, Langerwehe, Germany) and an adjusted flow rate of 12.5 mL/min.

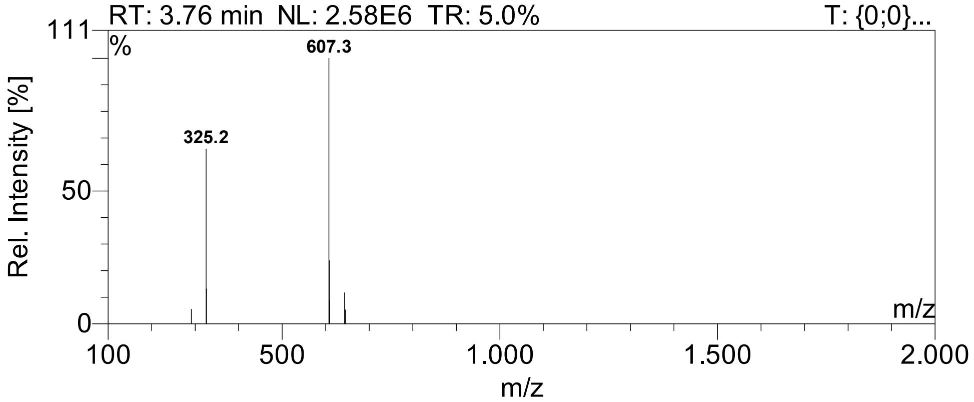


**Figure S3.** Mass spectrum of LacNAc-squarate monoamide ester. Electrospray ionization HPLC-ESI-MS and quadrupole mass analyzer (Finnigan Surveyor MSQ Plus, Thermo Scientific, needle voltage = 4 kV, temperature = 400 °C, cone voltage = 100 V, negative mode). The observed $[M-H]^-$ of 607.3 *m/z* corresponds well to the theoretical value of $[M-H]^-$ = 607.2 *m/z*.

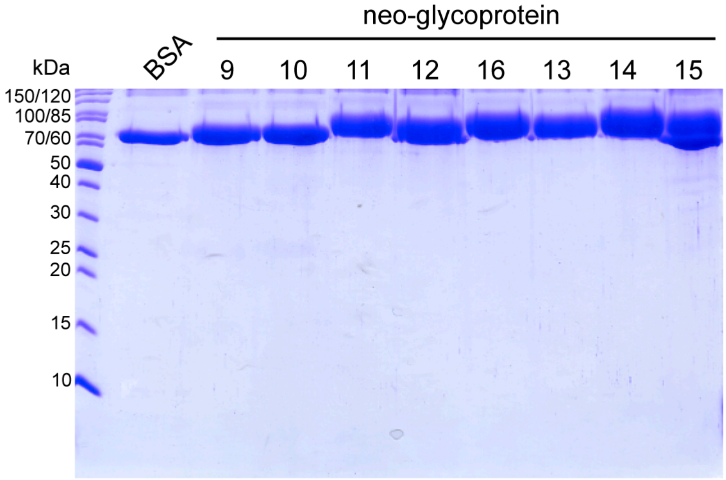


**Figure S4.** SDS-PAGE analysis of non-modified BSA and BSA neo-glycoproteins **9**–**16** carrying multiple glycan residues; M – PageRuler Prestained Protein Ladder (ThermoFisher Scientific). The increase of molecular weight and smearing character illustrates glycan attachment.

**Galectin-3 production**

The $His_6$-tagged human galectin-3 (Gal-3) was prepared as described previously [7]. Briefly, the gene for Gal-3 was amplified from human mesangial cDNA using the primers 5′-ATGGCAGACAATTTTTCGCTCCATGATGCG-3′and 5′-TATATCATGGTATATGAAGCACTGGTGAGGTC-3′. After introduction of restriction sites for SgsI and EcoR1 using the primers 5′-GGCGCGCCTTATATCATGGTATATGAAGC-3′ and 5′-CCGGAATTCCGGGATGGCAGACAATTTT-3′, the insert was ligated into the plasmid pETDuet-1 (Novagen, Merck, Darmstadt, Germany). The protein was recombinantly expressed in *E. coli* Rosetta (DE3) pLysS cells. Cultivation was performed in LB medium supplemented with appropriate antibiotics (130 rpm, 37 °C, 100 µg/mL ampicillin, 34 µg/mL chloramphenicol). On the next day, the one-liter main culture (TB medium, 5 L baffled flask, supplemented with respective antibiotics) was inoculated (1:10) and incubated at 37 °C (80 rpm) until an optical density ($OD_{600\ nm}$) of 0.5–0.8 was reached. The addition of isopropyl 1-thio-β-D-galactopyranoside (IPTG, 0.5 mM) induced protein expression. After 24 h post-induction, the cells were harvested by centrifugation (7,000 rpm, 30 min, 4 °C). For galectin purification, bacteria were suspended and sonicated on ice (two cycles, 30 s each, 52% amplitude). After removal of cell debris by centrifugation (15,000 rpm, 30 min, 4 °C), supernatant was filtered through 0.8 µm syringe filter. HisTrap™ HP 5 mL columns were used (GE Healthcare) according to manufacturer’s instructions for galectin-3 purification. Eluted Gal-3 was dialyzed against phosphate buffered saline (PBS, 50 mM NaH2PO4, 150 mM NaCl, pH 7.5) supplemented with 2 mM ethylenediaminetetraacetic acid (EPBS) using SnakeSkin™ Dialysis Tubing (10 kDa MWCO, ThermoFisher Scientific).

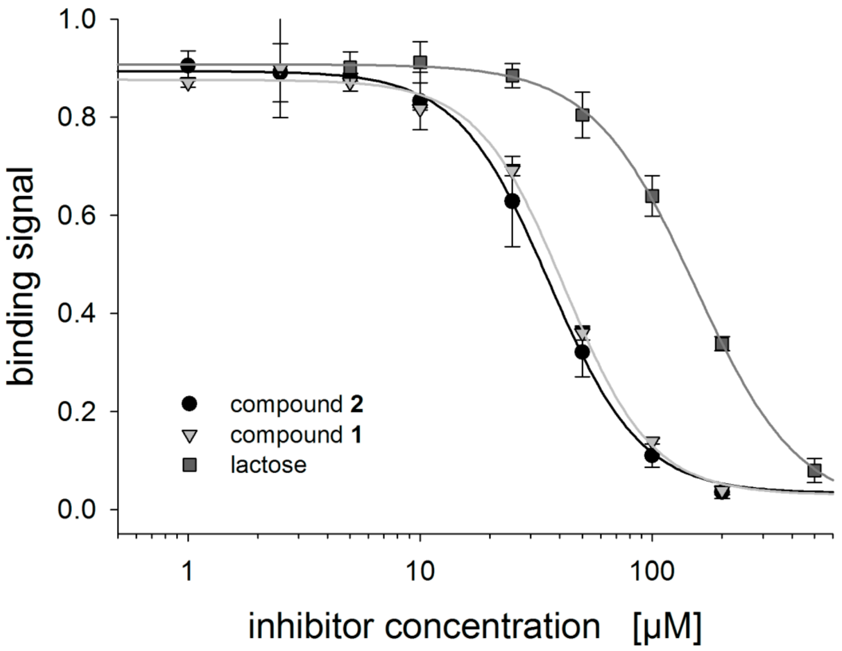


**Figure S5.** Competitive inhibition of Gal-3 binding to asialofetuin (ASF, coating amount: 5 pmol, 50 µL [0.1 µM] per well) using monovalent LacdiNAc (**1**), LacNAc (**2**) and lactose. Refer to Schemes S1 and S2 for compound composition. The respective $IC_{50}$ values are shown in Table 1.

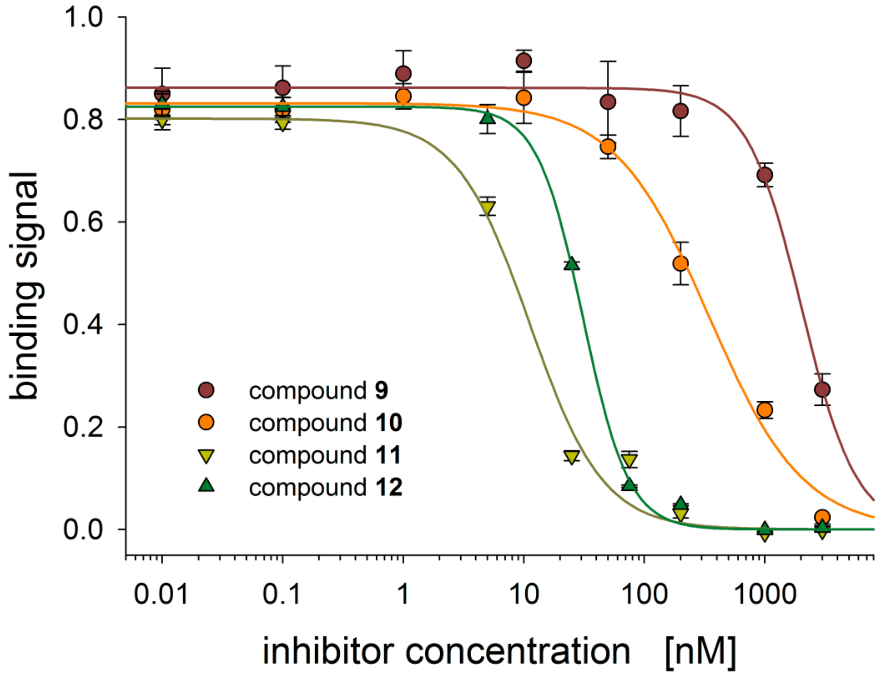


**Figure S6.** Inhibition of Gal-3 binding to immobilized ASF using BSA neo-glycoproteins **9**-**12** as inhibitors at indicated concentrations. Refer to Scheme S1 for compound composition. The respective IC50 values are listed in Table 1 in the main text.

**Table S1.** Comparison of binding affinities of His6-tag Gal-3 and Gal-3-AVI in ELISA assays

| Galectin-3 construct | $K_{d\ app}$ [μM], direct ELISA | IC50 glycan [μM], competitive ELISA | |
|---|---|---|---|
| | ASF | Lactose | LacdiNAc |
| $His_6$-tag Gal-3 | 5 ± 1 | 137 ± 27 | 42 ± 2 |
| Gal-3-AVI | 4.8 ± 0.2 | 110 ± 4 | 43 ± 4 |

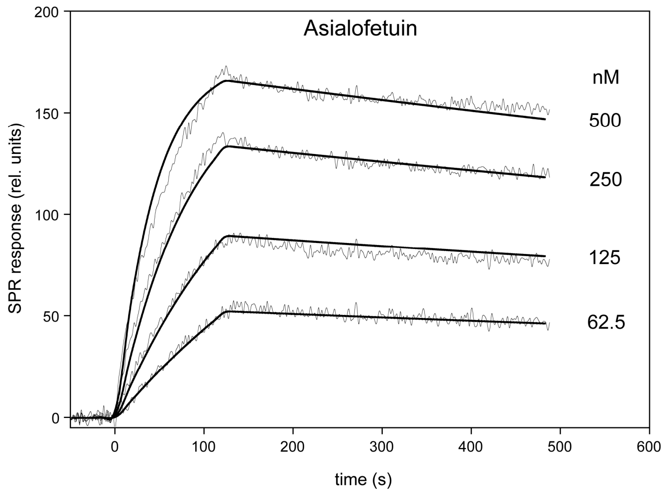


**Figure S7.** SPR kinetic binding analysis of the interactions between asialofetuin (ASF) and immobilized Gal-3-AVI. ASF was injected in parallel over the neutravidin-coated sensor chip coated with the biotinylated Gal-3-AVI at a flow rate of 30 μl/min. The kinetic data were globally fitted by using a 1 : 1 Langmuir binding model. The fitted curves are superimposed as thin black line on top of the sensograms.

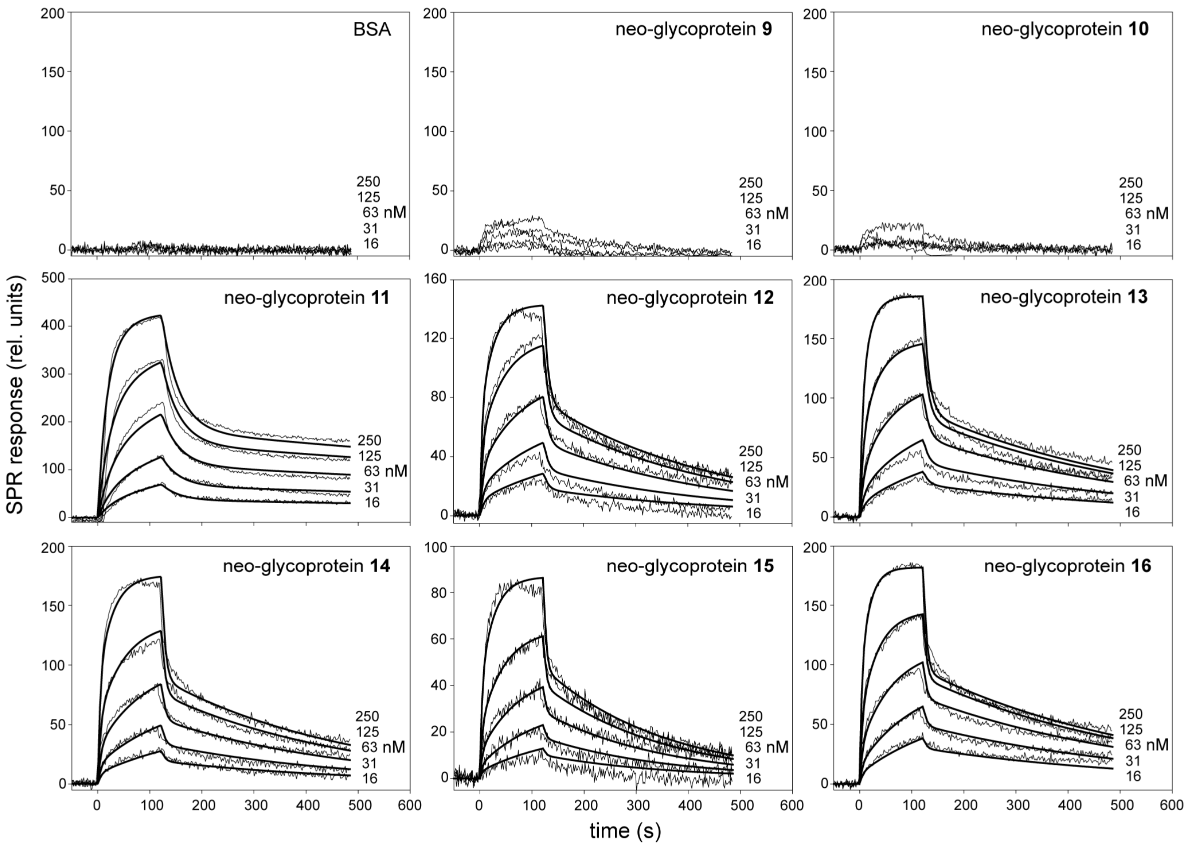


**Figure S8.** SPR kinetic binding analysis of the interactions between Gal-3 and immobilized neo-glycoproteins **9**-**16**. The fitted curves (heterogenous ligand model) are superimposed on top of the sensograms.

**Table S2.** Kinetic and binding affinity constants for the interactions of Gal-3 with immobilized neo-glycoproteins **9**-**16.**

| Compound | $k_{a1}$ [$M^{-1}$ $s^{-1}$] | $k_{d1}$ [$s^{-1}$] | $K_{D1}$ [M] | $k_{a2}$ [$M^{-1}$ $s^{-1}$] | $k_{d2}$ [$s^{-1}$] | $K_{D2}$ [M] |
|---|---|---|---|---|---|---|
| **9** | N.D. | N.D. | N.D. | N.D. | N.D. | N.D. |
| **10** | N.D. | N.D. | N.D. | N.D. | N.D. | N.D. |
| **11** | (1.2 ± 0.8) ×10$^{5}$ | (4.5 ± 2.1) ×10$^{-2}$ | (3.8 ± 1.9) ×10$^{-7}$ | (1.0 ± 1.1) ×10$^{5}$ | (9.4 ± 3.8) ×10$^{-4}$ | (9.4 ± 3.3) ×10$^{-9}$ |
| **12** | (9.3 ± 4.3) ×10$^{5}$ | (1.3 ± 1.1) ×10$^{-1}$ | (1.4 ± 0.9) ×10$^{-7}$ | (1.5 ± 1.2) ×10$^{5}$ | (2.9 ± 1.2) ×10$^{-3}$ | (1.9 ± 0.8) ×10$^{-8}$ |
| **13** | (4.1 ± 2.5) ×10$^{5}$ | (2.1 ± 1.1) ×10$^{-1}$ | (5.1 ± 3.0) ×10$^{-7}$ | (1.8 ± 1.2) ×10$^{5}$ | (2.1 ± 1.3) ×10$^{-3}$ | (1.2 ± 0.9) ×10$^{-8}$ |
| **14** | (3.3 ± 1.7) ×10$^{5}$ | (1.6 ± 1.2) ×10$^{-1}$ | (4.8 ± 2.6) ×10$^{-7}$ | (1.5 ± 1.0) ×10$^{5}$ | (2.7 ± 1.2) ×10$^{-3}$ | (1.8 ± 1.1) ×10$^{-8}$ |
| **15** | (3.9 ± 2.1) ×10$^{5}$ | (2.4 ± 1.3) ×10$^{-1}$ | (6.1 ± 3.1) ×10$^{-7}$ | (1.9 ± 1.5) ×10$^{5}$ | (4.1 ± 1.9) ×10$^{-3}$ | (2.2 ± 1.3) ×10$^{-8}$ |
| **16** | (3.5 ± 1.9) ×10$^{5}$ | (1.8 ± 1.4) ×10$^{-1}$ | (5.1 ± 2.8) ×10$^{-7}$ | (2.1 ± 1.4) ×10$^{5}$ | (2.3 ± 1.1) ×10$^{-3}$ | (1.1 ± 0.9) ×10$^{-8}$ |

## References


1. Blanchard, H.; Yu, X.; Collins, P.M.; Bum-Erdene, K. Galectin-3 inhibitors: a patent review (2008–present). *Expert Opin. Ther. Pat*. 2014, 24, 1053–1065, DOI:10.1517/13543776.2014.947961.
2. Collins, P.M.; Bum-Erdene, K.; Yu, X.; Blanchard, H. Galectin-3 interactions with glycosphingolipids. *J. Mol. Biol*. **2014**, *426*, 1439–1451, DOI:10.1016/j.jmb.2013.12.004.
3. Laaf, D.; Bojarová, P.; Pelantová, H.; Křen, V.; Elling, L. Tailored multivalent neo-glycoproteins: Synthesis, evaluation, and application of a library of galectin-3-binding glycan ligands. *Bioconjug. Chem*. **2017**, *28*, 2832–2840, DOI:10.1021/acs.bioconjchem.7b00520.
4. Böcker, S.; Laaf, D.; Elling, L. Galectin binding to neo-glycoproteins: LacDiNAc conjugated BSA as ligand for human galectin-3. *Biomolecules* **2015**, *5*, 1671–1696, DOI:10.3390/biom5031671.
5. Fischöder, T.; Laaf, D.; Dey, C.; Elling, L. Enzymatic synthesis of *N*-acetyllactosamine (LacNAc) type 1 oligomers and characterization as multivalent galectin ligands. *Molecules* **2017**, *22*, E1320, DOI:10.3390/molecules22081320.
6. Laaf, D.; Bojarová, P.; Mikulová, B.; Pelantová, H.; Křen, V.; Elling L. Two-step enzymatic synthesis of -D-*N*-acetylgalactosamine-(1→4)-D-*N*-acetylglucosamine (LacdiNAc) chitooligomers for deciphering galectin binding behavior. *Adv. Synth. Catal*. **2017**, *359*, 2101–2108, DOI:10.1002/adsc.201700331.
7. Kupper, C.E.; Rosencrantz, R.R.; Henssen, B.; Pelantová, H.; Thönes, S.; Drozdová, A.; Křen, V.; Elling, L. Chemo-enzymatic modification of *poly-N*-acetyllactosamine (LacNAc) oligomers and *N*,*N*-diacetyllactosamine (LacDINAc) based on galactose oxidase treatment. *Beilstein J. Org. Chem*. **2012**, *8*, 712–725, DOI:10.3762/bjoc.8.80.
